# Lipids of Marine Algae—Biomolecules with High Nutritional Value and Important Bioactive Properties

**DOI:** 10.3390/biom12010134

**Published:** 2022-01-14

**Authors:** M. Rosário Domingues, Ricardo Calado

**Affiliations:** 1 Centre for Environmental and Marine Studies (CESAM), Department of Chemistry, Campus Universitário de Santiago, University of Aveiro, 3810-193 Aveiro, Portugal; 2 Mass Spectrometry Centre & LAQV-REQUIMTE, Department of Chemistry, Campus Universitário de Santiago, University of Aveiro, 3810-193 Aveiro, Portugal; 3 ECOMARE, CESAM, Department of Biology, Campus Universitário de Santiago, University of Aveiro, 3810-193 Aveiro, Portugal

Marine microalgae are a multitude of taxonomically diverse unicellular organisms, ranging from diatoms to dinoflagellates and several other well-known groups, that may dwell in the water column, occur in marine sediments, or even associate symbiotically with marine animals. These organisms are a well-known source of lipids with high nutritional value and with important bioactive properties. Among these biomolecules, one can highlight *n*-6 and *n*-3 polyunsaturated fatty acids (PUFAs), glycolipids, and phospholipids. Macroalgae, also termed as seaweeds, are less diverse than microalgae and are currently divided in three different phyla: Chlorophyta (or green algae), Ochrophyta (or brown algae) and Rodophyta (or red algae). More recently, the lipids of macroalgae have also started to receive growing interest from the scientific community, likely a consequence of the remarkable growth that seaweed aquaculture has experienced worldwide. Indeed, there is an increasing awareness on how these “blue foods” may significantly contribute to enhance food security worldwide and how much have they been overlooked by researchers and policy makers as a valuable source of important nutrients such as lipids [1,2]. It is now unquestionable that the sustainable and innovative use of marine algae will be paramount to successfully achieve some of the United Nations Sustainable Development Goals (SDG), such as SDG 2 (Zero Hunger), SDG 13 (Climate Action) and SDG 14 (Life Below Water).

While the analysis and characterization of lipid content and fatty acid profiles has been surveyed for several decades in marine micro- and macroalgae, the lipidome of these taxa is only recently starting to be unraveled. The polar lipidome of marine algae is unanimously recognized as a rather untapped source of promising and valuable phytochemicals for multiple high-end applications, from food and feed to pharmaceutical, nutraceutical and cosmeceutical uses. Polar lipids are the main carriers of *n*-3 fatty acids, namely PUFAs, and have been reported to display anti-inflammatory, antioxidant, anti-microbial, and anti-proliferative properties.

Lipid signatures, from FA profiles to the in-depth profiling of lipid classes and species using the omics toolbox, reveal remarkable algal adaptations to shifting environmental conditions, reflecting changes in light intensity and spectra, seawater temperature and salinity, among several other drivers. Such unique fingerprints can support the development of origin certification protocols and can also be used as reliable proxies for the quality control of raw algae or algal-based products.

Overall, the accurate identification of lipid diversity in marine algae can foster the development of innovative algal-based products for multiple applications. Marine macroalgae will continue to be a strong driver of the blue bioeconomy, powered by biotechnological breakthroughs [3], and shading this new blue in green, red, and brown.

The scope of this Special Issue of *Biomolecules*, “Lipids of Marine Algae—Biomolecules with High Nutritional Value and Important Bioactive Properties”, was to provide an updated overview on the screening and use of lipids in marine algae. Seven contributions were published in this Special Issue, namely six original research articles and one review, addressing multiple issues featuring lipids of marine micro and macroalgae.

Remize et al. [4] performed isotopic labelling using pure ^13^C-CO_2_ gas to shed light over the pathways responsible for the synthesis of PUFAs 22:6*n*-3 and 20:5*n*-3 in the toxic dinophyte microalga *Alexandrium minutum*. This microalga is known to be responsible for harmful blooms (commonly termed as red tides) that may cause mortality among marine organisms. The isotopic labelling was followed in 11 FA to better understand synthesis pathways. The authors concluded that the polyketide synthase (PKS) pathway appeared to be a particularly fast synthetic process and that the enrichment dynamics of C18 PUFAs suggest that it is unlikely that these biomolecules are involved in the further desaturation and elongation steps of *n*-3 C20-C22 PUFAs.

Vigor et al. [5] profiled isoprostanoids using liquid chromatography-mass spectrometry/mass spectrometry (LC-MS/MS) in four marine microalgae (the diatoms *Chaetoceros gracilis* and *Phaeodactylum tricornutum*, the cryptophyte *Rhodomonas salina* and the haptophyte *Tisochrysis lutea*). Isoprostanoids are high-value bioactive metabolites resulting from the nonenzymatic oxidation of PUFAs (termed NEO-PUFAs). Additionally, the authors also monitored isoprostanoid profiles of microalgae under oxidative stress promoted by an exposure to copper and hydrogen peroxide. While the authors reported no significant variations in the content of oxidized derivatives in *R. salina* and *C. gracilis* exposed to copper, there was an increase in the production of C18-, C20- and C22-derived isoprostanoids in *T. lutea* and *P. tricornutum*. Concerning the exposure to hydrogen peroxide, no significant changes were observed for *C. gracilis* or *T. lutea*, while relevant variations were recorded for *P. tricornutum* and *R. salina*, namely for α-linolenic acid (C18:3*n*-3)-oxidized derivatives. The authors also highlighted how the manipulation of culture conditions holds the potential to enhance the production of isoprostanoids in target microalgae.

Remize et al. [6] characterized the synthesis pathways of the essential PUFA eicosapentaenoic acid (20:5*n*-3) (EPA) in the diatom *Chaetoceros muelleri* by monitoring the incorporation of ^13^C into 10 FA. The diatom rapidly incorporated ^13^C into C18 PUFAs, while EPA was one of the least enriched FAs. The authors suggested that the production of EPA by *C. muelleri* likely resulted from a combination of the *n*-3 (via 18:4*n*-3) and *n*-6 (via 18:3*n*-6 and 20:4*n*-6) synthesis pathways, with an alternative ω-3 pathway via 20:4*n*-6 probably also being involved. It is suggested that a structural and metabolic link may exist between 16:3*n*-4 and EPA in this and other diatoms, even if these two FA are not synthesized in the same cell compartments.

Patel et al. [7] reported the growth, lipid accumulation and FA profiles of the marine thraustochytrid *Aurantiochytrium* sp. T66 (ATCC-PRA-276) cultivated on six different volatile FAs (formic acid, acetic acid, propionic acid, butyric acid, valeric acid and caproic acid). Thraustochytrids are well known for being natural producers of *n*-3 FA, with up to 70% of total lipids they synthesize being docosahexaenoic acid (DHA) 22:6*n*-3. While the strain tested was unable to use propionic acid, valeric acid and caproic acid when these were provided at >2 g/L, it most efficiently used acetic acid and butyric acid when provided at up to 40 g/L. Indeed, when ATCC-PRA-276 was cultivated at 40 g/L butyric acid, the maximum DHA content recorded was 2.81 g/L, which corresponded to 42.63% *w*/*w* of total lipids. Another important finding reported by the authors was that fluorescence microscopy revealed that when cultivated with butyric acid, cell size increased up to 45 µm, a remarkably large value for oleaginous microorganisms, with numerous tiny lipid droplets also being visible in the microalgae cultivated under these conditions. This study paves the way for the potential use of volatile FA derived from side-streams of multiple industrial applications as cost-effective alternatives to pricey refined sugars commonly used as carbon sources.

da Costa et al. [8] employed high-performance liquid chromatography-mass spectrometry (HPLC-MS) to profile the lipidomic signatures of green seaweed *Ulva* spp. (popularly known as sea lettuces) harvested and cultivated from eight different geographic locations along the Iberian Peninsula Atlantic Coast. They hypothesized that lipidomic signatures could be successfully employed to trace the geographic origin of these valuable green seaweeds post-harvesting, whose collection and aquaculture is gaining momentum in Europe. Indeed, the lipidomic profile displayed by *Ulva* spp. originating from different locations differed significantly, namely due to the species of polyunsaturated betaine lipids and galactolipids recorded, along with those of saturated betaine lipids and sulfolipids and some phospholipid species as well. To refine their analysis, the authors selected a set of 25 site-specific molecular lipid species that allowed to assemble a unique lipidomic signature for geographic origin authentication and certification of *Ulva* spp. The plasticity of green seaweed lipidomes may be successfully used to fight fraudulent practices (e.g., the trading of cultivated seaweeds as wild caught or vice versa), as well as to allow a much-needed quality control of seaweed biomass being traded.

Monteiro et al. [9] determined the FA and lipidomic profiles of the brown seaweed *Saccharina latissima* (popularly known as sugar kelp) farmed offshore using long lines in three distinct locations in Europe, namely France, Norway, and the United Kingdom. Currently, this is the most heavily cultivated seaweed in Europe and the authors highlight that as the environmental conditions experienced by *S. latissima* in open sea culture sites are commonly unique at each location, these may shape the lipid profiles of cultured biomass and give origin to unique biochemical signatures. Seaweeds farmed at the northernmost location (Norway) displayed twice the lipid content recorded elsewhere, and those originating from the UK featured the lowest content of *n*-3 FA. The lipidomic profiles of *S. latissima* from France were enriched in lyso lipids, while those from Norway displayed a unique signature of phosphatidylglycerol, phosphatidylinositol, and phosphatidylcholine. Concerning seaweeds farmed in the UK, these featured higher levels of phosphatidylethanolamine and, in general, a lower content of galactolipids. Overall, the differences recorded made possible the identification of lipid species that are likely to represent origin biomarkers and can be used for purposes of traceability and food control.

The review by Harwood [10] brilliantly concludes this Special Issue of *Biomolecules* with an up-to-date description of very long-chain PUFA biosynthesis by algae. While fish oils remain the main sources of dietary EPA and DHA for human consumption, marine algae can produce these essential FA de novo. The author describes how these pathways can be successfully manipulated for commercial purposes, thus enhancing the potential of these organisms to foster a more sustainable blue bioeconomy. The drivers regulating the different pathways of very long-chain PUFA biosynthesis are also critically discussed so that cultivated algae can increasingly become a more economically feasible source of very long-chain PUFA. With demand for EPA and DHA peaking to unprecedented levels and not exhibiting any sign of slowing down in years to come, marine algae emerge as one of the most sustainable sources to deliver these much-needed biomolecules.

## Data Availability

Not applicable.
